# Supplementary material for: High Methoxyl Pectin and Sodium Caseinate Film Matrix Reinforced with Green Carbon Quantum Dots: Rheological and Mechanical Studies
Source: Membranes (Basel). 2022 Jul 7;12(7):695. doi: 10.3390/membranes12070695 (PMC9324415; doi:10.3390/membranes12070695)
Supplement: Supplementary file 1 [file membranes-12-00695-s001.zip › membranes-1794000-supplementary.pdf]

Supplementary Information

High Methoxyl Pectin and Sodium Caseinate Films Matrix Rein-Forced with Green Carbon Quantum Dots: Rheological and Mechanical Studies

Clarissa Murru [1], Mohammad Amin Mohammadifar [2], Jakob Birkedal Wagner [3], Rosana Badía Laiño [1], Marta Elena Díaz García [1]

[1] Departamento de Química Física y Analítica, Universidad de Oviedo, Oviedo, Asturias, 33006, Spain

[2] Research Group for Food Production Engineering, National Food Institute, Technical University of Denmark, SøltoftsPlads, 2800 Kongens Lyngby, Denmark

[3] DTU Nanolab, Technical University of Denmark, Fysikvej, DK-2800 Kgs. Lyngby, Denmark

Figure S1. Viscosity flow curves (log-log plot) of CAS:HMP suspensions (25:75 v/v ratio, pH 3.81 ± 0.4) in the absence and presence of APCDs measured at 25ºC. Inset table: Flow parameter

•CAS/HMP, •CAS/HMP+0.25%APCDs, •CAS/HMP+0.5%APCDs,•CAS/HMP+1.0%APCDs.

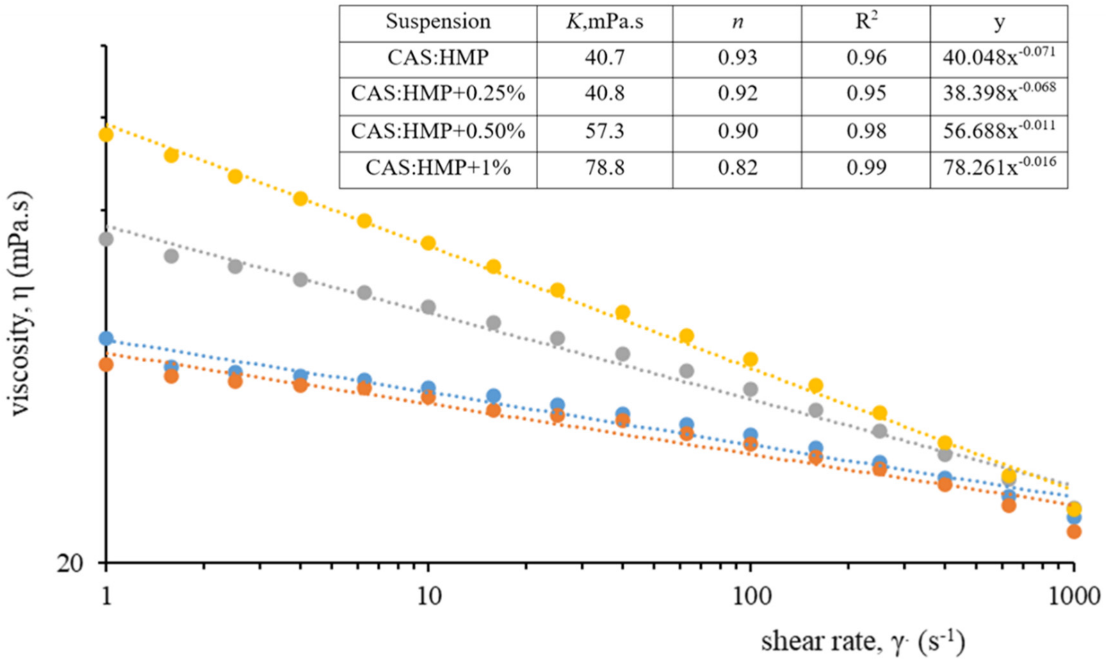

Suspension	*K*,mPa.s	*n*	R^2	y
CAS:HMP	40.7	0.93	0.96	$40.048x^{-0.071}$
CAS:HMP+0.25%	40.8	0.92	0.95	$38.398x^{-0.068}$
CAS:HMP+0.50%	57.3	0.90	0.98	$56.688x^{-0.011}$
CAS:HMP+1%	78.8	0.82	0.99	$78.261x^{-0.016}$

Figure S2. DSC thermograms of CAS /HMP films reinforced with APCDs

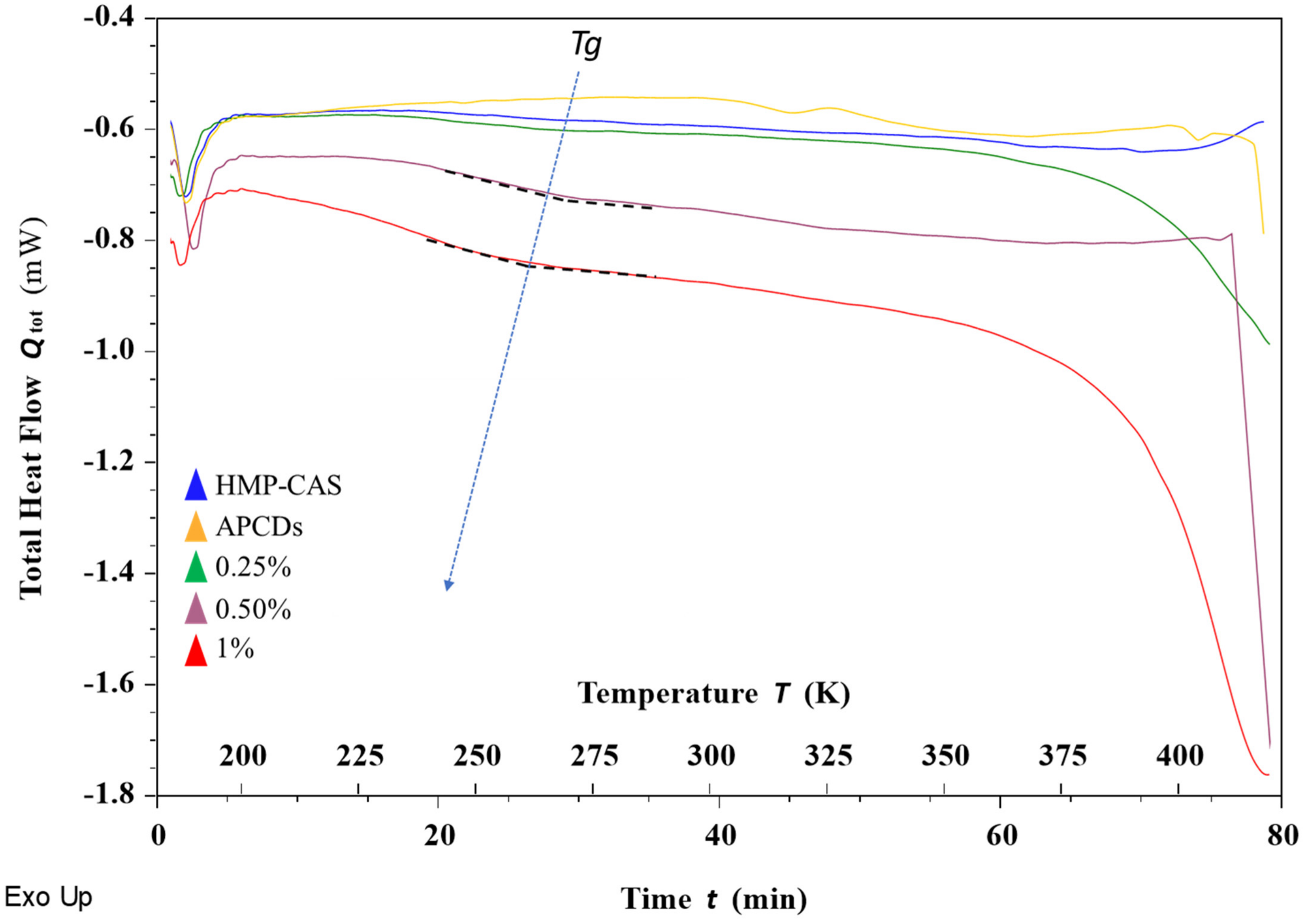

Figure S3. Complex modulus versus frequency in double logarithmic scales for CAS/HMP suspensions containing different amounts of APCDs: 0% (—), 0.25% (—),0.5% (—)and 1.0% (—).

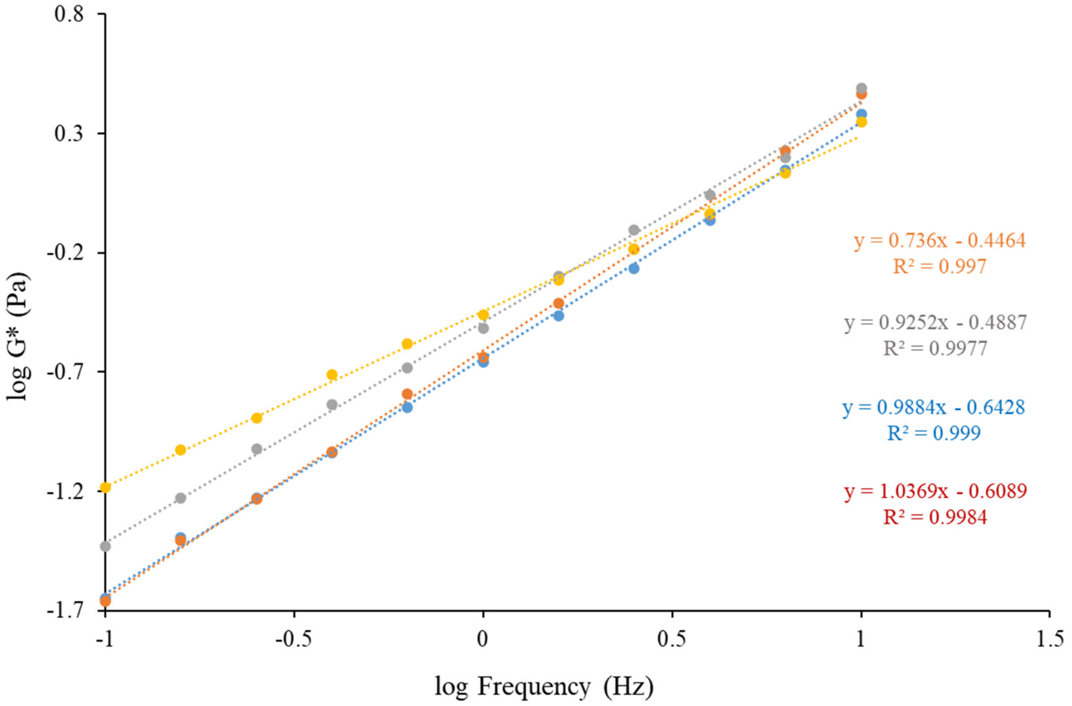

Figure S4. Loss tangent versus frequency for CAS/HMP suspensions containing different amounts of APCDs: 0% (—), 0.25% (—), 0.5% (—)and 1.0% (—).

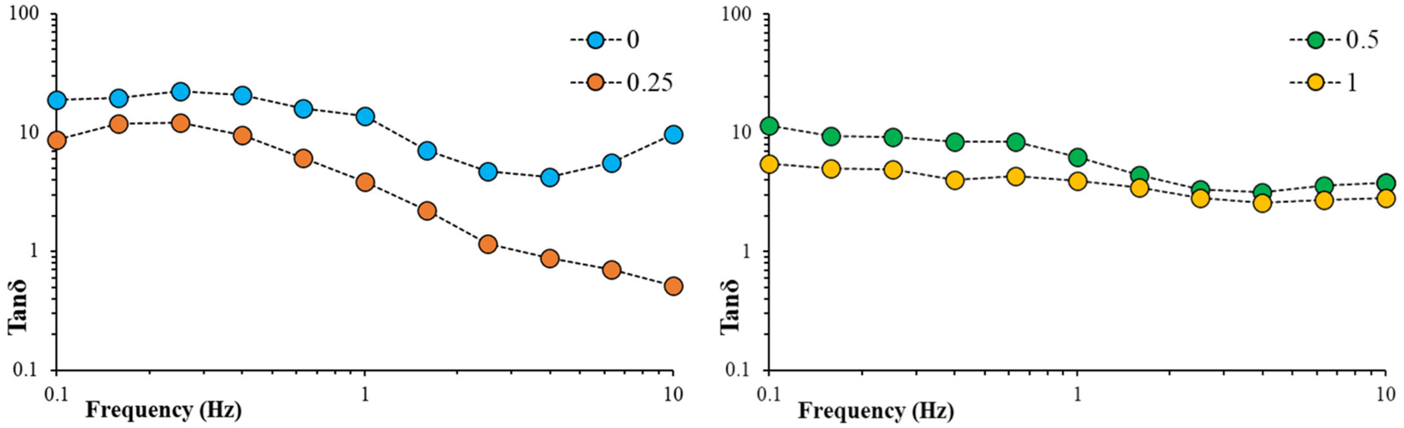

Figure S5. Rheological behaviour as a function of temperature and relative humidity (●10% RH, ●50%RH, ●70%RH.) of *bio*films CAS/HMP+1.0APCDs.

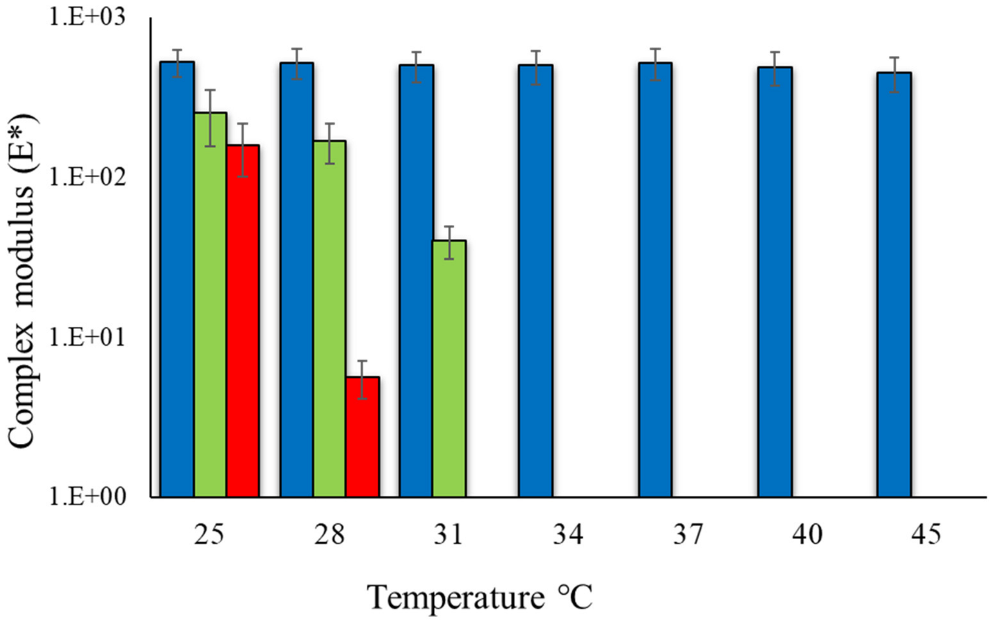

Figure S6. Rheological behaviour of biofilms as a function of exposure time at different temperatures. ● CAS/HMP, ● CAS/HMP+0.25APCDs, ● CAS/HMP+0.5APCDs, ●

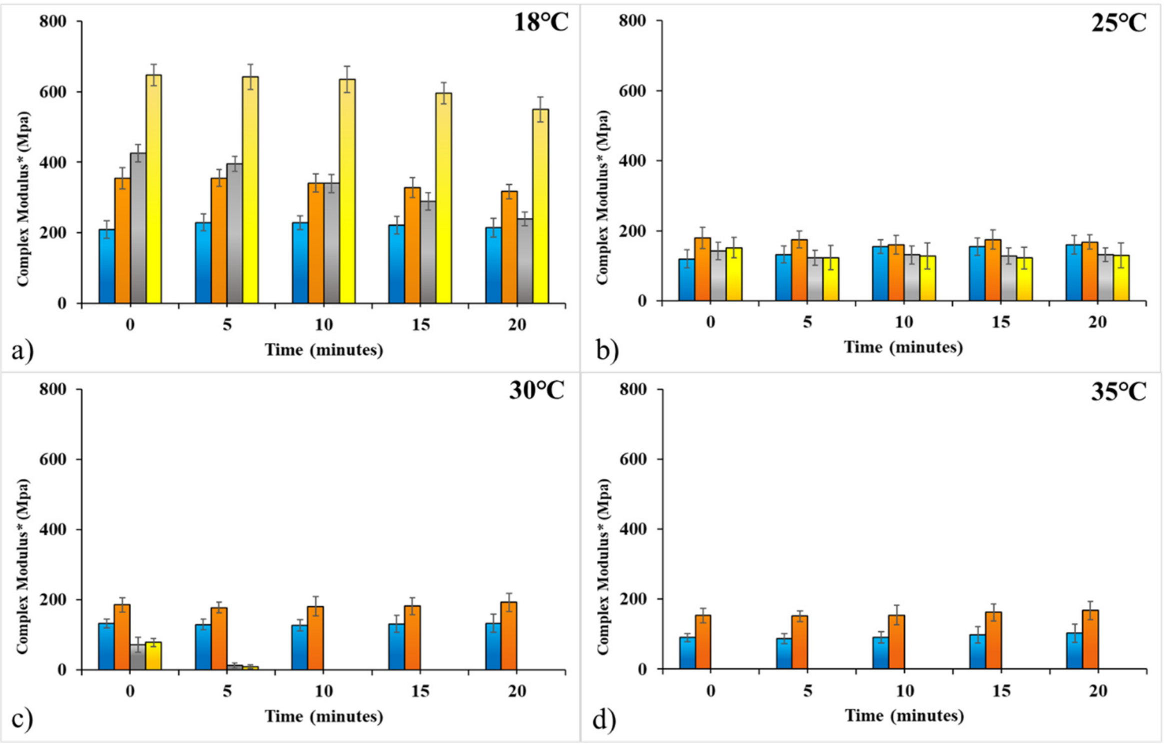

Filename: membranes-1794000-supplementary.docx
Directory: E:\7.7\pharmaceuticals-1793104
Template: C:\Users\MDPI\AppData\Roaming\Microsoft\Templates\Normal.dotm
Title:
Subject:
Author: clarissa murru
Keywords:
Comments:
Creation Date: 4/3/2022 9:43:00 PM
Change Number: 24
Last Saved On: 6/30/2022 9:47:00 PM
Last Saved By: clarissa murru
Total Editing Time: 3 Minutes
Last Printed On: 7/7/2022 3:38:00 PM
As of Last Complete Printing
Number of Pages: 4
Number of Words: 245 (approx.)
Number of Characters: 1,414 (approx.)
